# Radiofrequency ablation reduces expression of SELF by upregulating the expression of microRNA-26a/b in the treatment of atrial fibrillation

**DOI:** 10.1007/s10840-022-01305-x

**Published:** 2022-07-21

**Authors:** Min Dai, Tao Jiang, Cai-dong Luo, Wei Du, Min Wang, Qing-yan Qiu, Hu Wang

**Affiliations:** Mianyang Central Hospital, School of Medicine, University of Electronic Science and Technology of China, 12 Changjia Alley, Fucheng District, Mianyang, 621000 Sichuan China

**Keywords:** Atrial fibrillation, Ablation, P-Selectin, miR-26a, miR-26b, Coagulation

## Abstract

**Background:**

In this study, we aimed to investigate the role of miR-26a and miR-26b in the management of AF.

**Methods:**

Real-time PCR was carried out to determine plasma microRNA expression in AF patients pre- and post-radiofrequency ablation. The correlation between the expression of SELP and miR-26a/miR-26b was also studied using luciferase assays to establish a miR-26a/miR-26b/SELP signaling pathway.

**Results:**

The relative expression of SELP reached its peak in pre-ablation AF ( +) patients, while ablation treatment reduced the expression of SELP in AF ( +) patients. Similarly, AF pigs showed dysregulation of miR-26a/b and SELP, thus verifying the involvement of miR-26a/b and SELP in AF. Meanwhile, the regulatory association between SELP and miR-26a/b was also investigated, and the results showed that the presence of pre-miR-26a/b increased the levels of miR-26a/b and inhibited the mRNA/protein expression of SELP. Finally, using bioinformatic tools and luciferase assays, SELP mRNA was confirmed as the target of miR-26a/b, which affected the effect of AF ablation treatment.

**Conclusions:**

RFA helped to restore circulating levels of miR-26, which were reduced in atrial fibrillation. Meanwhile, miR-26 is a potential cause for the elevated plasma levels of pro-thrombogenic SELP in that disease.

## Introduction

As a highly frequent condition featured by high mortality and morbidity, atrial fibrillation (AF) is triggered by the abnormal propagation of electrical signals in the atrium and is known to be progressive in nature and leads to an increased risk of stroke [1]. According to a previous report, the crude mortality rate of AF patients for all-cause death was 6.33%, which accounts for a 3.67-fold higher risk of all-cause death than the general population [2]. As a widely used technique in the treatment of AF, catheter ablation is carried out by delivering alternating electrical current into targeted tissues. And the operation of catheter ablation is invasive, and late recurrence may occur after acutely successful radiofrequency catheter ablation [3, 4]. In fact, catheter ablation has been shown to increase thrombogenicity and induce the denaturation of blood components, thus causing endothelial disruption, char, thrombus formation, and coagulum. It was demonstrated that the site of ablation catheter placement in the electrophysiologic procedure shows increased levels of d-dimer and thrombin-antithrombin III [5].

As a cellular adhesion factor in the lectin family, P-selectin (SELP) is primarily expressed on platelets and endothelial cells. By exerting an essential effect on the early phase of leukocyte adhesion and recruitment to the endothelium, SELP regulates the interaction between platelets and leukocytes [6]. Among various ligands of SELP, SELP glycoprotein ligand-1 (SELPLG) is the most extensively characterized and was shown to mediate the rolling of leukocytes [7]. Moreover, SELPLG and SELP participate in the onset of cardiovascular disorders [8, 9]. SELPLG and SELP can also trigger different inflammatory disorders such as multiple sclerosis. In fact, Piccio et al. suggested that the plasma expression of SELPLG and SELP can be used as potential biomarkers for evaluating the magnitude of platelet activation in a wide range of pathogenic conditions, including AF, rheumatic mitral stenosis, and ischemic heart disorder [10–13]. Nevertheless, many studies on AF have produced conflicting data. For example, some studies showed increased expression of SELP and soluble SELP on the surface of platelets collected from AF patients, especially from those showing pre-embolic and embolic symptoms, while other studies failed to make such observations [10, 12, 14, 15].

As a key mediator in gene regulation, microRNA (miRNA) has become a research hotspot in recent years. As a type of short single-strand RNA molecule containing about 20 nucleotides, miRNAs do not code for proteins yet still play essential regulatory roles in gene transcription and translation of eukaryotic cells. During the process of miRNA-induced gene silencing, it binds to the 3′-UTR of its target gene and leads to its translational suppression [16]. In fact, many miRNAs are involved in cancer development.

As an important miRNA, microRNA-26a has been studied extensively. The expression of miR-26 is decreased in samples of atrial tissues collected from AF patients [17]. *In vitro* studies also showed that the KCNJ2 gene (encoding the IK1 pore-forming subunit) is targeted by miR-26 [18]. In addition, the knockdown of miRNA-26 by siRNA in mice increased Kir2.1 and IK1 expression and increased the vulnerability of these mice to AF. On the contrary, overexpression of miRNA-26 can decrease the severity of AF by reducing the levels of Kir2.1 and IK1 protein expression, suggesting that miRNA-26 exerts a protective effect in AF [17].

Here, we investigated the role of miR-26a/b and their downstream targets in the ablation treatment of human AF subjects and AF pigs, so as to assess the value of using miR-26a/b as diagnostic and therapeutic markers in the management of AF [19–22].

## Methods

### Human sample collection

Twenty-five AF patients (the AF + group) and twenty-five healthy volunteers (the AF − group) were recruited from Mianyang Central Hospital. The baseline characteristics of all subjects, such as their age, gender, and blood pressure, were recorded and summarized. Moreover, 5 ml of peripheral blood were drawn from each subject under fasting to measure the expression of miR-26a/b and SELP. The institutional ethics committee has approved this study and all participants signed informed consent before the initiation of this study.

### Animal model

Twenty-four 15-week-old pigs were chosen to create an AF swine model following a previously published approach [23]. 3 groups were established: an AF group, an AF plus ablation group, and a sham group. The animals in the AF group were subjected to AF induction using an electrode, which was linked to a subcutaneous neuro-stimulator under the neck, inserted in the RA appendage. The AF was induced by pacing the RA for seven consecutive days (400 pulses/min). The animals in the control group were subject to similar operations but the neurostimulator was shut off throughout the entire study. For animals in the AF plus ablation group, the dose of radiation was 60 Gy, and P-32 wire was carried out for 30 consecutive days according to a standard procedure [24]. After the animals were euthanized, myocardial tissues and blood samples were harvested to measure miR-26a/b and SELP expression. The institutional ethics committee has approved the protocols of this study, and this study was performed in accordance with the guidelines for animal experiments according to the Guide for the Care and Use of Laboratory Animals.

### Real-time PCR

RNA was isolated using TRIzol reagent (Carlsbad, Invitrogen, CA). The quality of isolated RNA was determined based on its ratio of OD260/OD280. Subsequently, isolated RNA samples were reversely transcribed into cDNA according to the recommendation of an RT kit (Thermo Fisher Scientific, Waltham, MA) and an RT PCR machine (Bio-Rad, Hercules, CA). In the next step, the levels of miR-26a, miR-26b, and SELP were quantified using an ABI 7500 machine (ABI, Foster City, CA) and an SYBR kit (TaKaRa, Tokyo, Japan) in accordance with standard procedures. The levels of miR-26a, miR-26b, and SELP mRNA were calculated using cycle threshold values, and U6 and GAPDH were respectively used as internal reference genes for the expression of miR-26a/b and SELP mRNA.

### Cell culture

H9C2 and HCM cells were maintained in DMEM with 10% FBS at 37℃ and 5% CO_2_. Then, the cells were transfected with precursors of miR-26a and miR-26b by Lipofectamine 3000 (Invitrogen, Carlsbad, CA). After 48 h of transfection, the cells were collected to quantify SELP expression.

### Luciferase assay

Complementary miR-26a and miR-26b “seed sequences” were found in SELP 3′ UTR using bioinformatic analyses. Subsequently, the 3′ UTR of SELP was sub-cloned into a pcDNA3.1 vector (Promega, Madison, WI) to create wild-type plasmids for the 3′ UTR of SELP, while mutations were introduced to “seed sequences” of miR-26a and miR-26b via site-directed mutagenesis (QuikChange II, Stratagene, San Diego, CA) to create mutant plasmids for the 3′ UTR of SELP. Then, H9C2 and HCM cells were co-transfected with miR-26a or miR-26b and appropriate wild-type/mutant 3′ UTRs of SELP. After 48 h of transfection, the luciferase activity of transfected cells was quantified using a luciferase assay kit (Promega, Madison, WI).

### Western blot

Samples were lysed to collect total proteins, whose concentrations were evaluated using a BCA kit (Thermo Fisher Scientific, Waltham, MA). In the next step, the protein samples were resolved using 10% SDS-PAGE, blotted onto a PVDF membrane (Millipore, Burlington, MA), blocked with PBS containing 5% non-fat milk, incubated at 4℃ overnight with anti-SELP primary antibodies (Abcam, Cambridge, MA), incubated at room temperature for 1 h with HRP-tagged secondary IgG antibodies, developed by an ECL kit (Bio-Rad, Hercules, CA), and analyzed by a gel imaging system (Bio-Rad, Hercules, CA) to determine SELP expression.

### Immunohistochemistry (IHC) assay

Following a routine procedure, fixed tissue sections were blocked with 3% H_2_O_2_, incubated sequentially with primary and secondary anti-SELP antibodies, counter-stained with hematoxylin, differentiated by hydrochloric acid alcohol, dehydrated with gradient alcohol, and assessed using ImageJ software.

### Statistical analysis

SPSS 22.0 (IBM, Chicago, IL) was used for statistical analysis. All data was shown in average ± SD. Statistical comparisons were tested with one-way ANOVA. And *P* < 0.05 was indicative of significant differences.

## Results

### Expression of miR-26a, miR-26b, and SELP was deregulated in AF patients

**Fig. 1:**
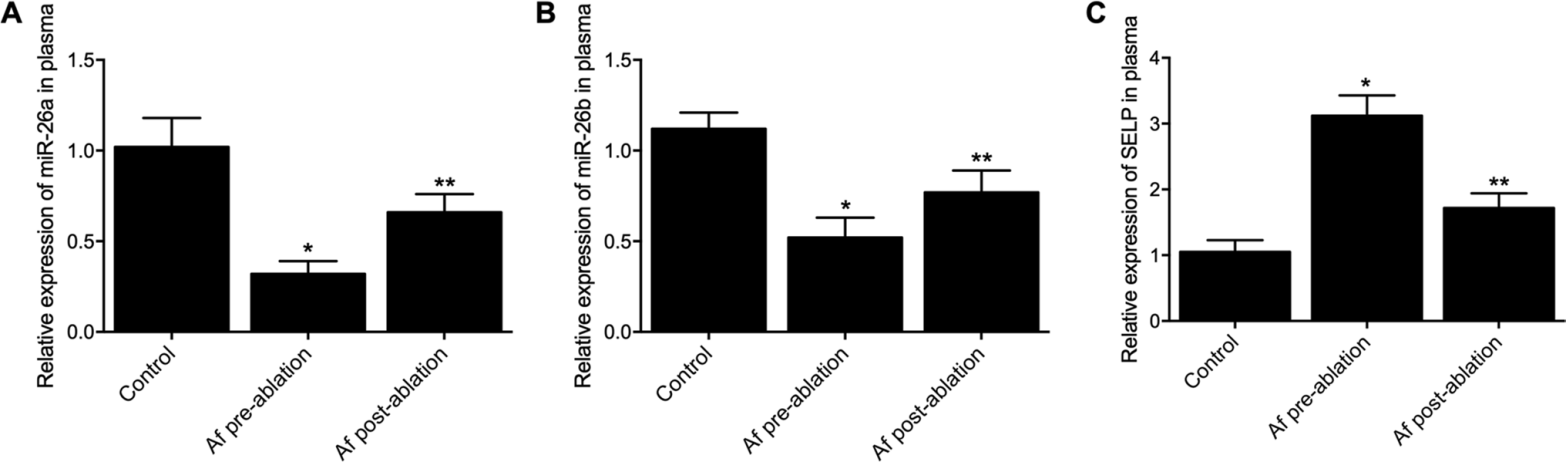
Plasma expression of SELP and miR-26a/b varied between AF ( +) and control groups. In addition, the ablation treatment also changed the plasma expression of SELP and miR-26a/b in AF patients (*n* = 25 in each group; *P value < 0.05 vs. control group; **P value < 0.05 vs. AF pre-ablation group). **A** Relative expression of plasma miR-26a was downregulated in the AF ( +) group, while the ablation treatment augmented the expression of miR-26a in the AF ( +) group to a certain extent; **B** relative expression of plasma miR-26b was downregulated in the AF ( +) group compared with that in the control group, while the ablation treatment augmented the expression of miR-26b in the AF ( +) group to a certain extent; **C** relative expression of plasma SELP was upregulated in the AF ( +) group compared with that in the control group, while the ablation treatment decreased the expression of SELP in the AF ( +) group to a certain extent

In this study, we recruited twenty-five AF patients (the AF ( +) group) and twenty-five healthy subjects (the control group). As shown in Table 1, the patients’ characteristics such as age and gender showed insignificant differences between the AF ( +) and control groups. Therefore, these characteristics were excluded from our analysis. The plasma levels of SELP and miR-26a/b in the control and AF ( +) groups were measured pre and post the ablation treatment. Accordingly, the plasma levels of miR-26a (Fig. 1A) and miR-26b (Fig. 1B) were decreased in the AF ( +) group. However, the ablation treatment augmented the expression of miR-26a/b in the AF ( +) group to a certain extent, although the miR-26a/b expression in post-ablation patients of AF was still lower than that in the control group. On the contrary, the relative expression of plasma SELP (Fig. 1C) peaked in AF patients before the ablation, and the ablation treatment reduced the expression of SELP to a certain degree. The overall expression of SELP was higher in AF patients than that in normal controls. Therefore, radiofrequency ablation could partially restore deregulated SELP and miR-26a/b expression in AF patients.

**Table 1:** Demographic and laboratory information of the enrolled subjects

Characteristics	Atrial fibrillation (*N* = 25)	Control (*N* = 25)	*P* value
Age (years)	53.5 ± 6.3	52.7 ± 8.8	0.7133
Male gender (%)	20 (80.0)	21 (84.0)	0.7156
Hypertension (%)	8 (32.0)	9 (36.0)	0.7676
Diabetes (%)	3 (12.0)	2 (8.0)	0.6407
Ejection fraction (%)	56.6 ± 7.5	55.3 ± 4.9	0.4716
Stroke/transient ischemic attack (%)	3 (12.0)	4 (16.0)	0.6407
Left atrial size (mm)	42.8 ± 3.7	43.1 ± 6.8	0.8472
Creatinine clearance (mL/min)	81.6 ± 27.5	78.8 ± 30.1	0.7328
D-dimer (µg/mL)	0.5 ± 0.4	0.6 ± 0.5	0.4387
BNP (pg/mL)	112.6 ± 135.1	109.5 ± 141.3	0.9371
APTT (s)^a^	48.9 ± 11.3	45.2 ± 15.7	0.3437
Hemoglobin	12.88 ± 1.05	13.84 ± 2.19	0.0539
Neutrophil	80.17 ± 11.23	61.33 ± 6.91	< 0.05
Lymphocyte	14.13 ± 8.48	32.73 ± 5.19	< 0.05
Neutrophil/lymphocyte ratio	5.77 ± 4.76	1.84 ± 0.73	< 0.05
Monocyte	5.93 ± 2.38	7.26 ± 2.05	< 0.05
Eosinophil	0.42 ± 1.05	1.73 ± 1.18	< 0.05
Basophil	0.48 ± 0.38	0.63 ± 0.31	0.1327
Mean platelet volume	8.46 ± 1.75	8.78 ± 1.33	0.4702
White blood cell count	10.65 ± 2.96	8.07 ± 1.28	< 0.05
Red cell distribution width	15.27 ± 0.73	15.61 ± 0.84	0.1332
C-reactive protein	13.99 ± 7.35	2.28 ± 0.83	< 0.05

### P-32 wire ablation in swine with AF restores pre-AF levels of miR-26a, miR-26b, and SELP

**Fig. 2:**
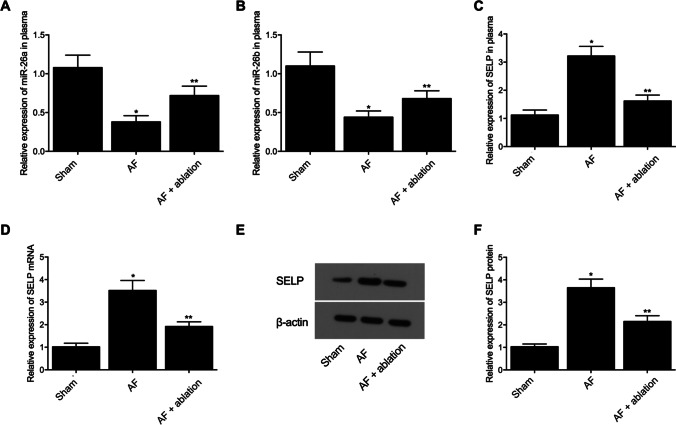
Expression of miR-26a, miR-26b, and SELP differed in pre-ablation AF pigs, post-ablation AF pigs, and sham-operated pigs, thus indicating that radiofrequency ablation helped the treatment of AF by suppressing the mRNA/protein expression of SELP in AF pigs (*n* = 8 in each group; *P value < 0.05 vs. Sham group; **P value < 0.05 vs. AF group). **A** Relative expression of plasma miR-26a was downregulated in the AF group compared with that in the sham group, while the ablation treatment augmented the expression of miR-26a in the AF group to a certain extent; **B** relative expression of plasma miR-26b was downregulated in the AF group compared with that in the sham group, while the ablation treatment augmented the expression of miR-26b in the AF group to a certain extent; **C** relative expression of plasma SELP mRNA was upregulated in the AF group compared with that in the sham group, while the ablation treatment decreased the expression of SELP mRNA in the AF group to a certain extent; **D** relative expression of SELP mRNA in myocardial samples was upregulated in the AF group compared with that in the sham group, while the ablation treatment decreased the expression of SELP mRNA in the AF group to a certain extent; **E** Western blot analysis was used to compare SELP protein expression in myocardial samples collected from the AF group (at both pre-ablation and post-ablation stages) and the sham group; **F** relative expression of SELP protein in myocardial samples was upregulated in the AF group compared with that in the sham group, while the ablation treatment decreased the expression of SELP protein in the AF group to a certain extent

**Fig. 3:**
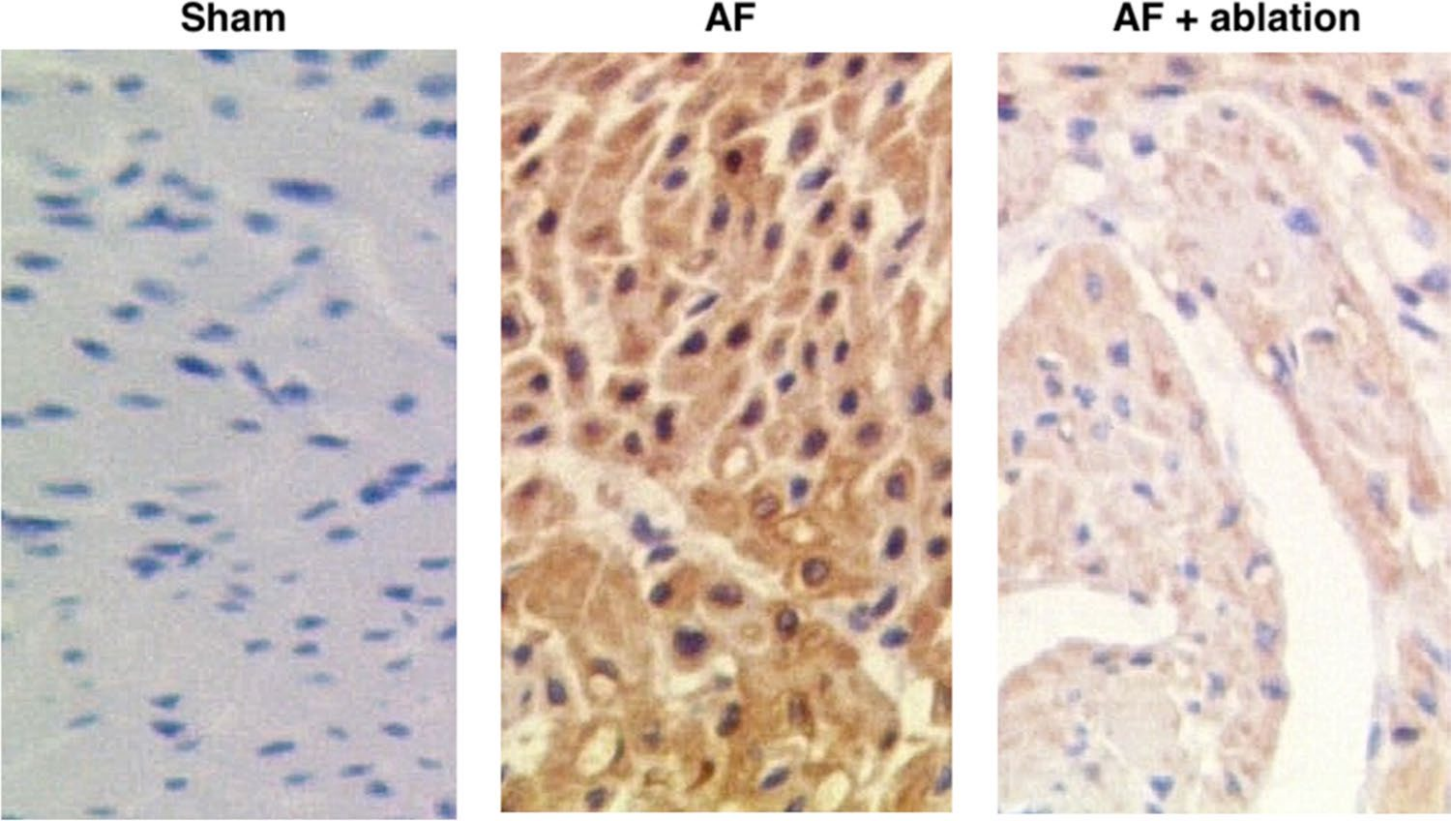
IHC assay showed that the relative expression of SELP protein was upregulated in the AF group compared with that in the sham group, while the ablation treatment decreased the expression of SELP protein in the AF group to a certain extent

Twenty-four 15-week-old pigs were chosen to create an AF swine model. Three groups were established: an AF group (*N* = 8), an AF + ablation group (*N* = 8), and a sham group (*N* = 8). Blood samples were then collected from AF pigs at pre-ablation and post-ablation stages, respectively, to measure their miR-26a/b and SELP expression. As shown in Fig. 2 and compared to the sham group, the plasma expression of miR-26a (Fig. 2A) and miR-26b (Fig. 2B) was decreased in AF pigs (at both pre-ablation and post-ablation stages) and along with increased expression of SELP mRNA (Fig. 2C). In addition, the treatment with ablation increased the plasma expression of miR-26a/b and decreased the plasma expression of SELP mRNA to a certain extent. Moreover, myocardial tissue samples were collected from the animals to measure their expression of SELP mRNA/protein. Compared with their levels in pre-ablation AF pigs, both the mRNA (Fig. 2D) and protein levels (Fig. 2E and F) of SELP were reduced in the myocardial tissue samples collected from post-ablation AF pigs, although the expression of SELP was the lowest in sham-operated pigs. Similar results were also obtained from IHC assays (Fig. 3), indicating that radiofrequency ablation suppressed SELP expression in P-32 wire ablation in swine.

### The negative regulatory relationship between SELP and miR-26a/b expression

**Fig. 4:**
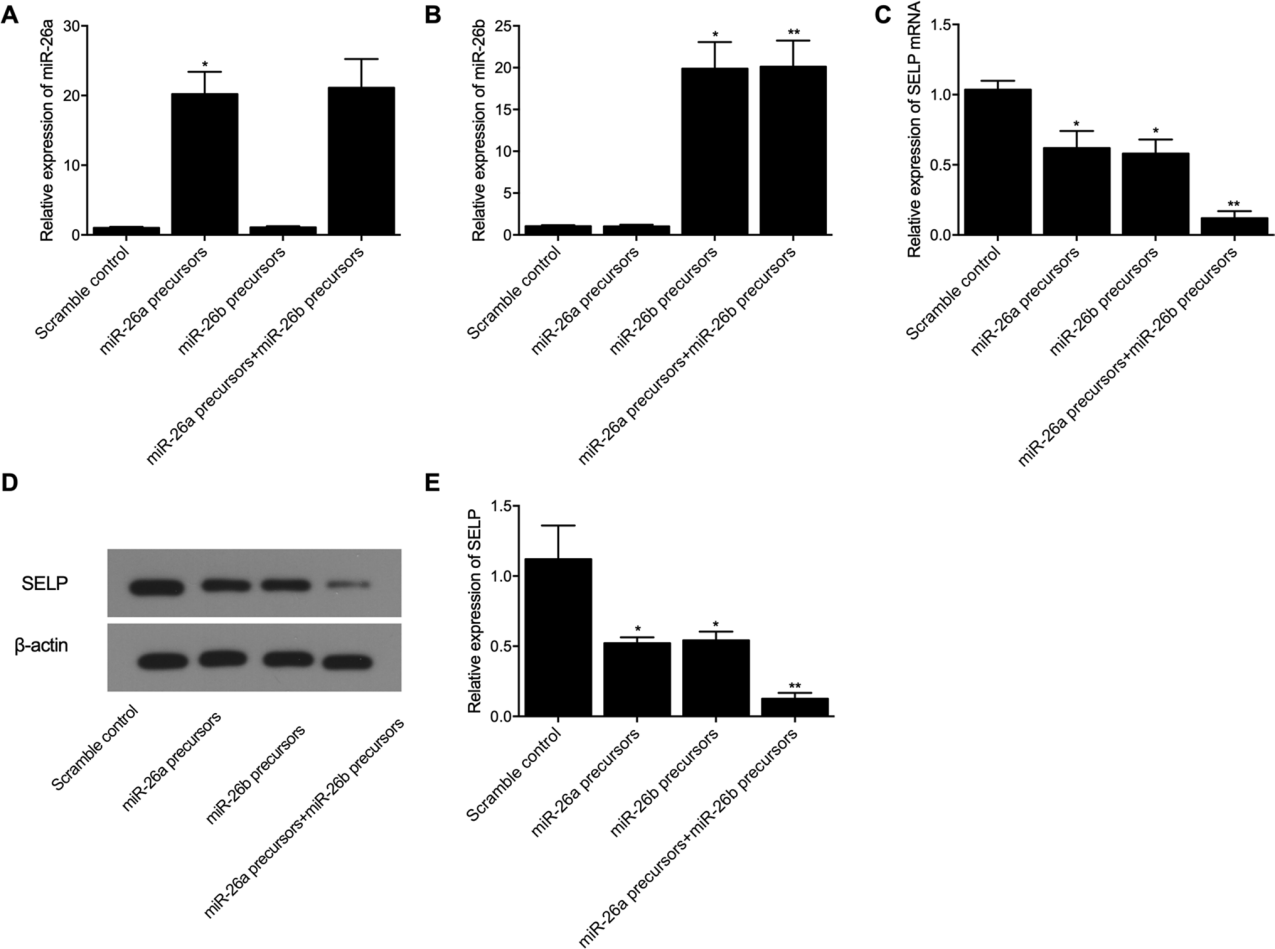
MiR-26a and miR-26b were shown to regulate the expression of SELP in HCM cells (*P value < 0.05 vs. scramble control; **P value < 0.05 vs. miR-26a precursors). **A** Expression of miR-26a was upregulated in HCM cells transfected with pre-miR-26a or pre-miR-26a + pre-miR-26b, while the transfection of cells with pre-miR-26b exerted no effect on the expression of miR-26a; **B** expression of miR-26b was upregulated in HCM cells transfected with pre-miR-26b or pre-miR-26a + pre-miR-26b, while the transfection of cells with pre-miR-26a exerted no effect on the expression of miR-26b; **C** compared with that in the control group, the expression of SELP mRNA was downregulated in HCM cells transfected with pre-miR-26a or pre-miR-26b, and the transfection of cells with both pre-miR-26a and pre-miR-26b most suppressed the mRNA expression of SELP; **D** Western blot analysis showed the expression of SELP protein in HCM cells transfected with pre-miR-26a, pre-miR-26b, or pre-miR-26a + pre-miR-26b; **E** compared with that in the control group, the expression of SELP protein was downregulated in HCM cells transfected with pre-miR-26a or pre-miR-26b, and the transfection of cells with both pre-miR-26a and pre-miR-26b most suppressed the protein expression of SELP

**Fig. 5:**
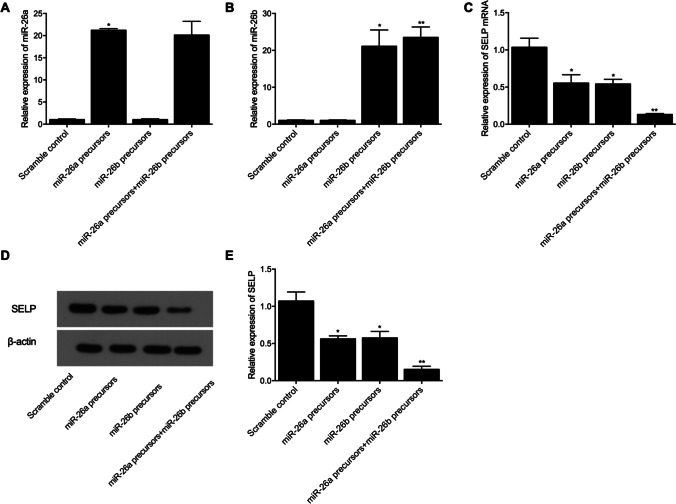
MiR-26a and miR-26b were shown to regulate the expression of SELP in H9C2 cells (*P value < 0.05 vs. scramble control; **P value < 0.05 vs. miR-26a precursors). **A** Expression of miR-26a was up-regulated in H9C2 cells transfected with pre-miR-26a or pre-miR-26a + pre-miR-26b, while the transfection of cells with pre-miR-26b exerted no effect on the expression of miR-26a; **B** expression of miR-26b was upregulated in H9C2 cells transfected with pre-miR-26b or pre-miR-26a + pre-miR-26b, while the transfection of cells with pre-miR-26a exerted no effect on the expression of miR-26b; **C** compared with that in the control group, the expression of SELP mRNA was downregulated in H9C2 cells transfected with pre-miR-26a or pre-miR-26b, and the transfection of cells with both pre-miR-26a and pre-miR-26b most suppressed the mRNA expression of SELP; **D** Western blot analysis showed the expression of SELP protein in H9C2 cells transfected with pre-miR-26a, pre-miR-26b, or pre-miR-26a + pre-miR-26b; **E** compared with that in the control group, the expression of SELP protein was downregulated in H9C2 cells transfected with pre-miR-26a or pre-miR-26b, and the transfection of cells with both pre-miR-26a and pre-miR-26b most suppressed the protein expression of SELP

We also studied the interaction between SELP and miR-26a/b in HCM and H9C2 cells transfected with miR-26a/b precursors. As shown in Fig. 4, the HCM cells were divided into a scramble control group, a pre-miR-26a group, a pre-miR-26b group, and a pre-miR-26a + pre-miR-26b group. The level of miR-26a (Fig. 4A) was upregulated in the pre-miR-26a and pre-miR-26a + pre-miR-26b groups, while the relative expression of miR-26b (Fig. 4B) was also upregulated in the pre-miR-26b and pre-miR-26a + pre-miR-26b groups. Furthermore, the transfection of HCM cells with pre-miR-26a/b suppressed the relative expression of SELP mRNA (Fig. 4C) and protein (Fig. 4D and E), while the co-transfection of HCM cells with both pre-miR-26a/b most reduced SELP expression. When the above experiments were repeated in H9C2 cells (Fig. 5), similar results were obtained. Therefore, it can be concluded that, although no interaction was observed between miR-26a/b, the upregulation of miR-26a/b expression could downregulate the expression of SELP mRNA/protein, while the presence of both miR-26a/b could most suppress the expression of SELP mRNA/protein.

### Both miR-26a/b targeted SELP

**Fig. 6:**
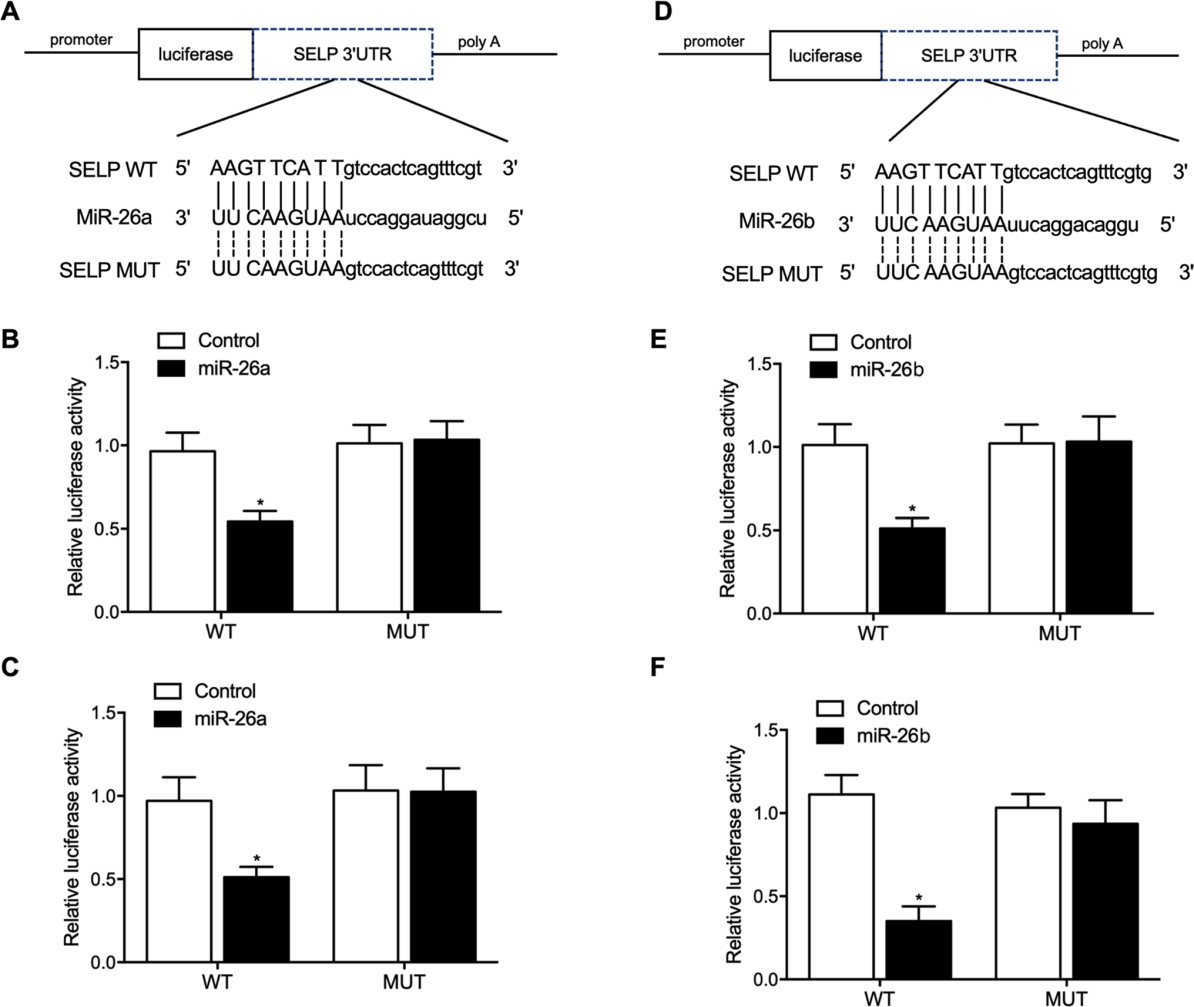
Both miR-26a and miR-26b targeted SELP (*P value < 0.05 vs. control). **A** A potential “seed sequence” of miR-26a was identified in SELP mRNA; **B** luciferase activity was reduced in HCM cells co-transfected with miR-26a and wild-type SELP mRNA; **C** luciferase activity was reduced in H9C2 cells co-transfected with miR-26a and wild-type SELP mRNA; **D** a potential “seed sequence” of miR-26b was identified in SELP mRNA; **E** luciferase activity was reduced in HCM cells co-transfected with miR-26b and wild-type SELP mRNA; **F** luciferase activity was reduced in H9C2 cells co-transfected with miR-26b and wild-type SELP mRNA

Two candidate “seed sequences” of miR-26a/b were found on the 3′UTR of SELP mRNA and matched the sequences of miR-26a (Fig. 6A) and miR-26b (Fig. 6D), respectively. Compared with the luciferase activity in other cell groups, the luciferase activity of HCM and H9C2 cells was decreased after co-transfection with wild-type SELP mRNA and miR-26a mimics (Fig. 6B  and C) or miR-26b mimics (Fig. 6E and F), indicating that SELP mRNA acts as a virtual target of miR-26a/b.

## Discussion

As a major burden on public health, AF is associated with an increasing incidence as the world population becomes older. As a pro-inflammatory and pro-thrombotic disorder, AF can cause significant morbidity and mortality due to the onset of severe cerebrovascular and cardiovascular complications. In recent years, the use of catheter ablation to relieve the symptoms of AF has become quite popular, although in general multiple catheter ablation, procedures are needed to completely alleviate the symptoms of AF [25]. In this study, miR-26a/b expression was elevated in post-ablation AF patients. In addition, the plasma levels of miR-26a/b were inhibited in the AF ( +) group, along with elevated expression of SELP. Furthermore, the ablation treatment helped to increase the plasma levels of miR-26a/b while decreasing the level of SELP in the AF ( +) group to a certain extent. Therefore, radiofrequency ablation could partially restore deregulated miR-26a/b and SELP expression in AF patients.

It was shown that miR-26 might play a potential and novel regulatory role in AF. Furthermore, miR-26b also regulates the functions of many DEGs and has been found to play important roles in cardiovascular disorders. Based on the results from functional analysis, miRNAs can control some biological processes, including the metabolic processes of glucan and energy as well as the development of blood and heart vessels, during the progression of AF. Some miRNAs, including miR-355p and miR-26b, have been implicated in AF-induced inflammatory reactions. In a previous study, it was shown that some DEGs, including TPM3, and miRNAs, including miR-26b and miR-355p, act as hubs, indicating that the interaction of miRNA-DEGs also plays a critical role in AF-induced inflammation. In a recent study, the overexpression of miRNA-26 was found to decrease the severity of AF by reducing the protein levels of Kir2.1 and IK1, suggesting a protective effect of miRNA-26 in AF [17]. The authors of this study also showed that miRNA-26 expression in a POAF group was downregulated compared with that in patients with sinus rhythm. According to the findings, the overexpression of miR-26 decreases the vulnerability to AF, and the downregulation of miR-26 expression contributes to the development of post-CABG POAF [17]. In this study, compared with that in sham-operated pigs, we found that the relative expression of plasma miR-26a/b was decreased, but the relative expression of plasma SELP mRNA was increased in AF pigs. In addition, the ablation treatment increased miR-26a/b expression but reduced the plasma level of SELP mRNA in AF pigs. Moreover, the mRNA/protein expression of SELP in myocardial tissue samples showed a similar trend as that in blood samples. Therefore, it can be concluded that radiofrequency ablation exerts a beneficial effect on the treatment of AF by partially restoring the dysregulated mRNA/protein expression of SELP in AF.

In another study carried out using an animal model of AF, a successful procedure of ablation in AF treatment was featured by a low rate of thromboembolic events. In fact, in most patients whose symptoms of AF are completely relieved by ablation, the application of oral anti-coagulants can be discontinued without increasing the likelihood of thromboembolic incidents and bleeding. The lack of thromboembolic events in these patients indicates that if patients with a high risk of symptomatic events are monitored closely, the application of oral anti-coagulants may be safely discontinued. In fact, it is widely known that a correlation is present between the outcome of catheter ablation and the expression of inflammation markers in the circulation [26–28]. Recently, it was shown that a successful procedure of AF ablation can change the profiles of inflammatory markers and affect the chance of AF recurrence following the procedure of AF ablation [27, 29]. Some studies have investigated ablation-induced changes in the profiles of inflammatory biomarkers. For example, it was shown that the amount of P-selectin, a factor representing the level of platelet activation, is elevated in cardiac circulation following the onset of AF [30], and decreased miR-26 level was reported to be associated with increased platelet activation in the sepsis [31]. In this study, when the cells were transfected with pre-miR-26a, pre-miR-26b, or pre-miR-26a plus pre-miR-26b, the levels of miR-26a/b were increased, respectively. Meanwhile, the transfection of cells by pre-miR-26a or pre-miR-26b both inhibited the mRNA/protein expression of SELP, while the co-transfection of cells with both pre-miR-26a/b most inhibited SELP expression. Therefore, it can be concluded that the upregulation in miR-26a/b expression downregulates the mRNA/protein expression of SELP.

As an adhesion molecule mediating the rolling of leukocytes 6, SELP was demonstrated to induce atherogenesis. Moreover, preferential expression of SELP was observed in the endothelium underlying the formation of atherosclerotic plaques [31]. In addition, mice with SELP deficiency tend to develop decreased fatty streaks, while antibodies against SELP could inhibit monocyte rolling and monocyte attachment to the carotid endothelium [32–35]. Furthermore, atherogenic mediators, such as oxidized LDL, were shown to induce SELP expression [36, 37].

The delivery of circulating microparticles to thrombi via PSGL-1 and SELP seems to be important in the normal accumulation of tissue factors and the generation of fibrin in thrombi. Accumulating evidence has demonstrated the role of SELP and PSGL-1, a major ligand of SELP, in the accumulation of tissue factors and subsequent synthesis of fibrin in the platelet thrombus. Nevertheless, the blockage of SELP activities by corresponding antibodies suppressed the accumulation of fibrin by almost 70% in the developing thrombi [38]. SELP can also mediate the adhesion between platelets and neutrophils to enhance the intracellular signaling in neutrophils, promote the release of superoxide anions from neutrophils, and facilitate the synthesis of leukotriene C4 and thromboxane [39]. In an animal experiment, Minamino et al. showed that the rapid pacing of atrial tissues augmented SELP expression [40]. In addition, AF patients also showed an increased level of baseline SELP.

## Conclusion

In summary, this study not only demonstrated the regulatory relationship between miR-26a/b and SELP but also validated that the radiofrequency ablation treatment augmented miR-26a/b expression and thus suppressed the expression of SELP.

## Data Availability

The data that support the findings of this study are available from the corresponding author upon reasonable request.
